# Evaluation of prognostic histological parameters proposed for pleural mesothelioma in diffuse malignant peritoneal mesothelioma. A short report

**DOI:** 10.1186/s13000-021-01125-z

**Published:** 2021-07-22

**Authors:** Federica Pezzuto, Luigi Vimercati, Francesco Fortarezza, Andrea Marzullo, Antonio Pennella, Domenica Cavone, Alessandra Punzi, Concetta Caporusso, Antonio d’Amati, Teresa Lettini, Gabriella Serio

**Affiliations:** 1 Pathology Unit Department of Cardiac, Thoracic, Vascular Sciences and Public Health, University of Padova, via A. Gabelli 61, 35121 Padova, Italy; 2 Department of Interdisciplinary Medicine, Occupational Health Division, University of Bari, 1 Umberto I Sq., 70121 Bari, Italy; 3 Pathology Unit, Department of Emergency and Organ Transplantation – DETO, University of Bari, 1 Umberto I Sq., 70121 Bari, Italy; 4 Pathology Unit, Department of Surgery, University of Foggia, 121 Napoli St, 71122 Foggia, Italy; 5 Pathology Unit, IRCCS National Cancer Institute “Giovanni Paolo II”, 65 Orazio Flacco St, 70124 Bari, Italy

**Keywords:** Pleural mesothelioma, Peritoneal mesothelioma, Histology, Grading

## Abstract

**Introduction:**

Diffuse malignant peritoneal mesothelioma (DMPM) is a rare malignant neoplasm with poor survival that shares some similarities with the best-known pleural variant, pleural mesothelioma. The recent European Reference Network on Rare Adult Cancers (EURACAN)/International Association for the Study of Lung Cancer (IASLC) proposals attempted to improve the histological diagnosis and patient risk stratification. Herein, we investigated whether the pathology recommendations and suggestions of the pleural proposals were applicable to diffuse malignant peritoneal mesothelioma.

**Methods:**

Fifty multiple laparoscopic biopsies of DMPM were consecutively collected at the Pathology Unit of the University of Bari. A two-tier system, i.e., low, and high grade, was used to categorize 34 epithelioid DMPMs. Architectural patterns, cytological features and stromal changes were also reported. Immunohistochemistry was performed for BRCA1-associated protein 1 (BAP1), programmed death-ligand 1 (PD-L1), and Ki67, while fluorescence in situ hybridization (FISH) was performed for p16/cyclin-dependent kinase inhibitor 2A (*CDKN2A)*.

**Results:**

High-grade epithelioid mesothelioma, high Ki67, and *p16/CDKN2A* deletion were significantly associated with short survival (*p* = 0.004, *p* < 0.0001, and *p* = 0.002, respectively). BAP1 loss and PD-L1 negativity were the most common findings. Multivariate analysis revealed that the nuclear grading system and p16 deletion significantly correlated with survival (*p* = 0.003 each).

**Conclusions:**

The present study examined the prognostic significance of several factors proposed for pleural mesothelioma in an extra pleural site. Notably, the introduction of a grading system may provide better risk stratification in epithelioid DMPM. Ki67, BAP1 and *p16*/*CDKN2A* should also be measured whenever possible. A detailed report with all supportive data would allow us to collect sufficient information for use in further studies on larger case series.

## Introduction

Malignant mesothelioma is a rare, fatal malignancy that primarily affects the visceral pleura. Most studies have been performed on the pleural variant, from which some information on the peritoneal form has been extrapolated. Most mesotheliomas are etiologically attributable to environmental asbestos exposure [1, 2], but a certain genetic susceptibility has also been investigated [3]. Although several differences have been found between the two entities, pleural and peritoneal mesotheliomas share some similarities. Diffuse malignant peritoneal mesothelioma (DMPM) has a poor prognosis. Long-term survivors are rare and exceptionally described in the literature [4]. The median survival is shorter than 1 year if untreated, but treatment strategies are limited. Cytoreductive surgery and heated intraperitoneal chemotherapy are the primary therapies for resectable forms [5]. Because the advantages obtained in prognosis and treatment response remain limited, the development of sensitive/specific diagnostic systems and the identification of prognostic/predictive factors are a priority. Some recommendations for appropriate pathology reports and diagnoses in DMPM have been established recently [5]. Some histological and immunohistochemical factors have also been investigated as prognostic indicators [6]. The recent European Reference Network on Rare Adult Cancers (EURACAN)/International Association for the Study of Lung Cancer (IASLC) proposal [7] was a step toward a multidisciplinary approach to pleural mesothelioma to improve the diagnostic precision and patient risk stratification. We investigated whether the pathology recommendations and suggestions of the pleural proposal were applicable to DMPM. In particular, we focused on histological parameters with a prognostic implication in a broad sense that is histotype, prevalent architectural pattern, cytological and stromal features, nuclear grading, necrosis, BAP-1 expression and PD-L1 positivity. In addition, the proliferative index has also been considered, as strongly recommended by the last Peritoneal Surface Oncology Group International (PSOGI)/EURACAN clinical practice guidelines for DMPM.

## Materials and methods

The histological diagnosis of DMPM was confirmed on 50 multiple laparoscopic biopsies that were consecutively collected at the Pathology Unit of the University of Bari between March 1990 and December 2017. A single sample was evaluated for each patient. When multiple specimens were available, the most representative sample (in terms of neoplastic cell content) was chosen.

The local Ethics Committee of the Policlinic Hospital, Bari, Italy approved the study. A panel of antibodies was used for diagnosis [5]. Samples were classified as epithelioid, biphasic and sarcomatoid histological subtypes. The epithelioid variant was further evaluated for predominant architectural patterns (tubulopapillary, trabecular, adenomatoid, microcystic, solid, micropapillary, transitional, pleomorphic, when detected in greater than 50% of the tumor surface), cytological features (rhabdoid, deciduoid, small cell, clear cell, signet ring, lymphohistiocytoid), stromal characteristics (myxoid), nuclear atypia, mitotic count and the presence of necrosis [8]. Nuclear atypia was scored from 1 (mild) to 3 (severe). Mitotic count was assigned a score of 1, 2 or 3 based on mitoses in 1, 2–4 and ≥ 5 per 2 mm^2^, respectively. The nuclear grade was derived from the sum of the scores for atypia and mitotic count: sum of 2–3 was grade I; sum of 4–5 was grade II, and sum of 6 was grade III. Tumours were further categorized into low-grade tumors (tumors with nuclear grade I and nuclear grade II without necrosis) and high-grade tumors (tumors with nuclear grade II in the presence of necrosis and nuclear grade III).

Immunohistochemistry for Ki67 (MIB-1, clone K5001, DAKO) and BRCA1-associated protein 1 (BAP1) (C4, Santa Cruz Biotechnology, Santa Cruz, USA) was performed on all samples. Ki67 expression was expressed as the percentage of positive cells in the total cell number. A complete absence of nuclear staining was considered true negative BAP1 staining in the presence of nuclear-positive lymphocytes. Programmed death-ligand 1 (PD-L1) (clone 22C3, DAKO) was examined in 27 cases. P16/cyclin-dependent kinase inhibitor 2A (CDKN2A) deletion was also assessed using fluorescence in situ hybridization (FISH) analysis, as previously described [6].

Univariate analyses were performed using the non-parametric Mann-Whitney U or Kruskal-Wallis tests for continuous variables and Fisher’s exact test or chi-squared test for categorical variables. Intra-observer and interobserver data reproducibility were tested for mitotic count, Ki67 value and tumor grade by considering all cases recorded by two observers (FP and GS), one of whom recorded the series twice at different times. The observer variations and correlations were examined using paired t tests and Pearson’s correlation coefficient.

Survival was calculated from the day of the pathological diagnosis to death or last follow-up. The overall survival was calculated using the Kaplan-Meier method with log-rank analysis. Multiple linear regression analyses were also performed. Significance was two-tailed and set at 0.05.

## Results

**Fig. 1:**
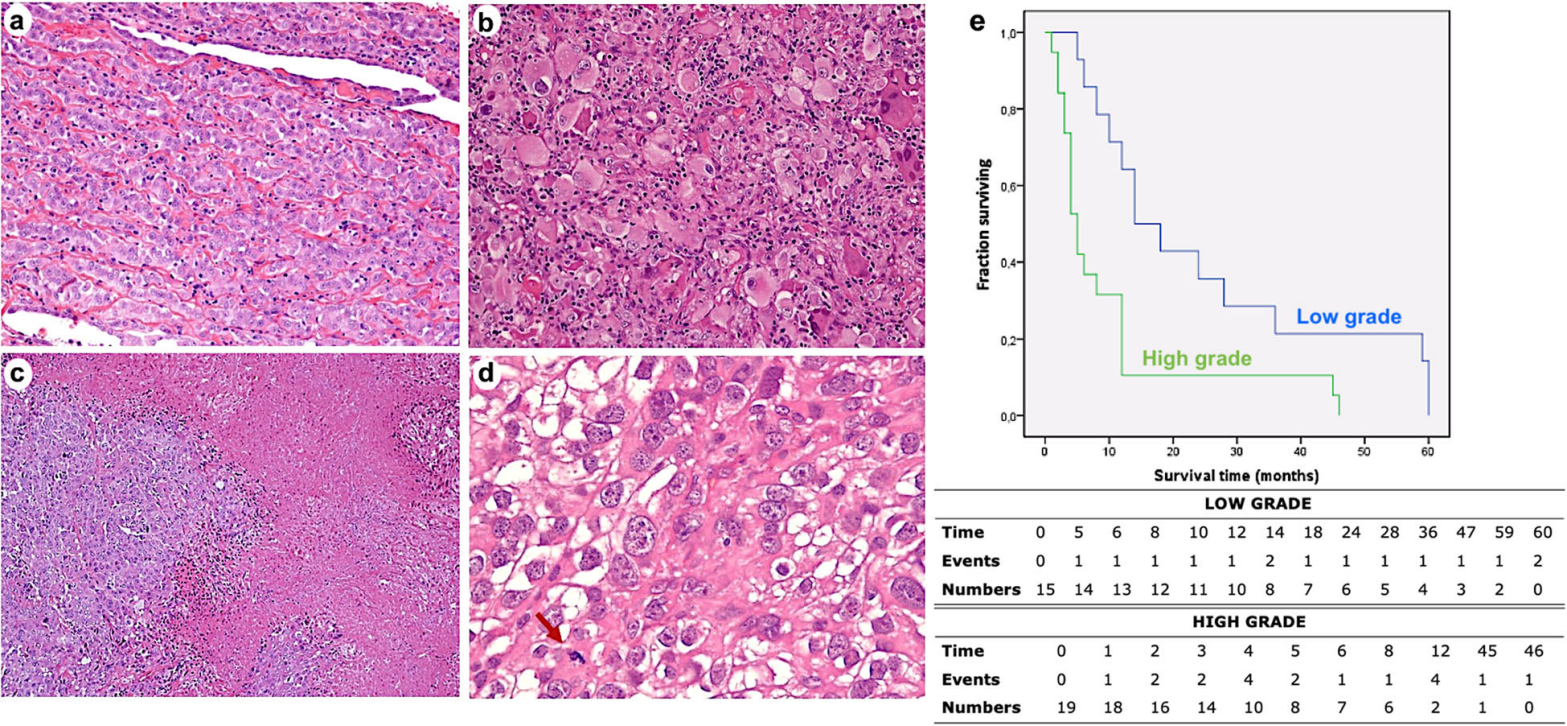
Low- and high-grade tumors. Representative images of low- (**a**, hematoxylin and eosin stain, original magnification ×100) and high-grade tumors with marked nuclear atypia (**b**, hematoxylin and eosin stain, original magnification ×200), foci of necrosis (**c**, hematoxylin and eosin stain, original magnification × 100) and high mitotic count (**d**, hematoxylin and eosin stain, original magnification × 400). A significant difference in survival was detected (p = 0.004) according to the tumor grade (**e**)

Fifty patients with a diagnosis of DMPM were included. The median age was 63 years (mean 63.8 ± 11.5; range 36–89 years). Thirteen patients were females (26%). Our series was quite homogeneous in treatment, although the data were collected over a long-time frame. All the patients were chemo-naïve at the time of biopsy. None of the patients underwent cytoreductive surgical therapy, intraperitoneal chemotherapy or immunotherapy after diagnosis. All patients had died at the last follow-up. There were 34 cases (68%) of epithelioid, 12 (24%) biphasic and 4 (8%) sarcomatoid histological subtypes (Table 1). A predominantly solid architectural pattern was most frequent in epithelioid mesotheliomas, and it was present in 25 (73%) cases. Six cases (18%) showed a desmoplastic stromal reaction, and 1 (3%) case showed myxoid changes. Nuclear atypia 2 and 3 were present in 16 and 18 cases, respectively. The median mitotic count was 5 per 2 mm^2^ (mean 6.4 ± 5.3; range 1–29). Foci of necrosis were evident in 12 (35.3%) cases. Epithelioid mesotheliomas were classified into low grade (Fig. 1a, b) and high grade (Fig. 1c, d) and detected in 15 and 19 cases, respectively (Table 2). The median percentage of Ki67 was 25.5%. BAP1 loss was found in 43 cases (86%). PD-L1 was positive in one (2%) biphasic case. The *p16/CDKN2A* homozygous deletion was detected in 30 (60%) cases (Table 1). High-grade tumors showed significantly different Ki67 values than low-grade tumors (mean 28.9 ± 12,8, CI 95% 22.7–35.1; median 26 vs. mean 16.1 ± 13.7, CI 95% 8.5–23.7, median 13.5; *p* = 0.009). For the reproducibility test, the t test was not significant in all cases. The correlation coefficient was r = .987 for the interobserver comparison of Ki67, and this coefficient was significant (*p* = 0.03).

**Table. 1:** Histologic subtypes, immunohistochemical and molecular features of diffuse malignant peritoneal mesothelioma

**Histological subtypes [N (%)]**	
‑ Epithelioid	34 (68%)
‑ Biphasic	12 (24%)
‑ Sarcomatoid	4 (8%)
**CDKN2A status [N (%)]**	
‑ Homozygous deletion	30 (60%)
‑ Heterozygous deletion	12 (24%)
‑ No deletion	8 (15%)
**BAP1 loss [N (%)]**	43 (86%)
**PD-L1 (tumor proportion score) [N (%)]**	
‑ < 1%	40 (98%)
‑ 1–49%	1 (2%)
‑ ≥ 50%	0
**Ki67 [%, median (Q1-Q3)]**	25.5 (15.25–41)

**Table. 2:** Morphological features of diffuse malignant epithelioid peritoneal mesothelioma

Architectural pattern	N (%)
‑ Tubulopapillary	6 (18%)
‑ Trabecular	1 (3%)
‑ Microcystic	1 (3%)
‑ Solid	25 (73%)
‑ Micropapillary	1 (3%)
**Cytological features**	
‑ Epithelioid	28 (82%)
‑ Deciduoid	6 (18%)
**Stromal features**	
‑ Non reactive	27 (79%)
‑ Desmoplastic	6 (18%)
‑ Myxoid	1 (3%)
**Nuclear atypia**	
‑ 1	0
‑ 2	16 (47%)
‑ 3	18 (53%)
**Mitotic count**	
‑ 1 (≤ 1/2 mm^2^)	1 (3%)
‑ 2 (2–4/2 mm^2^)	15 (44%)
‑ 3 (≥ 5/2 mm^2^)	18 (53%)
**Necrosis**	
‑ Present	12 (35%)
‑ Absent	22 (65%)
**Grade**	
‑ Low	15 (44%)
‑ High	19 (56%)

The median survival was 6 months (Q1-Q3: 3–14; mean 13.7 ± 16.9; CI 95% 9.04–18.4; range 1–60). Survival showed statistical significance for sex, and it was lower in females. A significant difference in survival was detected according to the histological subtypes, with the shortest survival observed in the sarcomatoid (*p* = 0.002) mesotheliomas, and in high-grade epithelioid tumors (*p* = 0.004) (Fig. 1e). A low Ki67 percentage, nuclear grade II, no deletion/heterozygous deletion of *p16/CDKN2A* showed the longest survival (*p* = 0.004, *p* = 0.0001 and *p* = 0.002, respectively). No statistical significance was found for BAP1 loss, even when a longer median survival was detected for cases with BAP1 alteration (8 months vs. 2 months). The small number of cases with each architectural pattern and stromal change did not allow reliable statistical correlations to be drawn. Survival data are reported in Table 3.

**Table. 3:** Survival data

Parameter	Mean survival (months)				Median survival (months)				***P***-Value
	CI 95%				CI 95%				
	Estimate	SE	Lower limit	Higher limit	Estimate	SE	Lower limit	Higher limit	
**Sex**									
**Males**	16.2	3.1	10.2	22.1	8	3	2.1	13.9	0.04*
**Females**	8.8	2.1	2.7	10.8	3	1.8	0	6.6	
**Age**									
**< 63 yrs**	11.4	3.3	5	17.8	5	1.5	2.1	7.9	**0.58**
**≥ 63 yrs**	16.1	3.5	9.2	22.9	12	2.7	6.7	17.3	
**Histotype**									
**Epithelioid**	17.4	3.2	11.2	23.6	10	2.2	5.7	14.3	
**Biphasic**	7	3	1.2	12.8	2	0.6	0.9	3.1	0.002*
**Sarcomatoid**	2.8	1	0.7	4.8	1				
**Tumor grading**									
**Low**	25.3	5.5	14.5	36	14	3.7	6.7	21.3	0.004*
**High**	10	3	4.1	15.9	5	0.7	3.6	6.4	
**Nuclear grading**									
**2**	27.8	5.2	17.6	37.9	18	10	0	37.6	< 0.0001*
**3**	8.2	2.3	3.6	12.7	5	0.7	3.6	6.4	
**Ki67**									
**Low**	24.1	4.3	15.8	32.5	14	2.7	8.8	19.2	
**High**	5.5	1.3	2.9	8.1	4	0.7	2.6	5.4	< 0.0001*
***p16/CDKN2A***									
**No deletion**	22	8.8	4.7	39.3	6	6.4	0	18.5	
**Heterozygous**	23.7	5.5	12.9	34.3	12	2.7	6.8	17.3	0.002*
**Homozygous**	7	1.5	4.1	10	4	0.9	2.3	5.8	

*Abbreviations*: *CI* Confidence interval; *SE* Standard error

*for statistical significance

Multiple linear regression analysis including Ki67 (both punctual values and cut-offs), nuclear grading, necrosis, and *p16/CDKN2A* deletion showed that only nuclear grading and *p16/CDKN2A* were significant predictors of survival (*p* = 0.003 each) (Table 4).

**Table. 4:** Multiple linear regression analysis

Parameter	Coefficient Table Iteration 1				Coefficient Table Iteration 2				Coefficient Table Iteration 3			
	Coeff	SE	Stand Coeff	***P***-value	Coeff	SE	Stand Coeff	***P***-value	Coeff	SE	Stand Coeff	***P***-value
**Nuclear grading**	−10.34	5.5	−0.32	***0.07***	−9.46	5.23	−0.29	***0.08***	−14.38	12.28	−0.44	0.003*
**Necrosis**	3.15	5.34	0.08	0.56	–	–	–	–	–	–	–	–
**Ki67 (low/high)**	−9.04	6.23	−0.24	0.15	−9.98	5.95	−0.27	0.10	–	–	–	–
***P16/CDKN2A*deletion**	−10.4	3.28	−0.43	***0.004***	−9.96	3.16	−0.04	***0.004***	−10.51	3.23	−0.44	0.003*

*Abbreviations*: *Coeff* Coefficient, *SE* Standard error, *Stand* Standard

*for statistical significance

## Discussion

The present study found that the grading system proposed for pleural malignant mesothelioma (MM) also significantly correlated with patient survival in DMPM. Several pathological grading systems have been proposed for pleural mesothelioma over the last decade [8–10]. However, none of these systems were standardized for DMPM. Valente et al. was the first study to combine nuclear features and mitoses for epithelioid DMPM [11] and showed a strong correlation with survival. A composite nuclear-grading system was successfully applied in a recent multi-institutional series of 225 cases [12]. Our results are consistent with this last study on the role of necrosis. Necrosis did not affect survival in our epithelioid case series when included in multivariate analysis. Our findings support the importance of establishing a standardized grading system to achieve better risk stratification in epithelioid DMPM. The application of the same method for all mesotheliomas, regardless of their origin, would be a step toward greater homogeneity in histological reports.

Other histological findings were associated with a distinct clinical outcome in our case series. Patients with epithelioid tumors had significantly longer survival than patients with other histological types. This result was not an unexpected finding because the current histological classification is a strong predictor of survival, with epithelioid MM having the best prognosis [13–15]. The epithelioid type was also the most frequently detected in our study population. This result is consistent with the literature [16]. Epithelioid MMs include a wide spectrum of tumors with variegated features that are associated with different clinical courses [7]. The clinical and pathological heterogeneity must be further characterized to identify pathological factors that may be associated with a more indolent tumor type or a poor outcome. The prognostic importance of architectural patterns and stromal reaction in DMPM are recognized [17]. even if concerns of the reproducibility of some histological parameters were raised [18]. We characterized the architectural and stromal features within our study population. However, our samples lacked some subgroups. Therefore, the evaluation of interobserver agreement and a comparison of survival were difficult to achieve, and evaluation should be postponed until a greater number of cases is available.

BAP1 expression was lost in most patients in our cohort. The complete loss of BAP1 expression was reported in a high percentage of MM [19], but never in reactive proliferation, which suggests the use of BAP1 as a highly specific method for differentiating MM and benign mesothelial proliferation [20]. BAP1 is only occasionally altered (0.3%) in serous ovary carcinoma, which further supports its utility in supporting a pathological diagnosis of peritoneal mesothelioma rather than gynecological carcinoma [21]. BAP1 inactivation altered the clinical outcome [19]. Protein nuclear expression is extremely feasible and may be considered a reliable marker for the complete loss of BAP1 activity [19]. BAP1 was only detected in 7 cases in our study population, which limited the power of statistical tests. Because the immunohistochemical evaluation of BAP1 is useful for diagnostic purposes and fairly specific for the diagnosis of MM in the appropriate histological context, it may be easily evaluated and inserted in the final report. Larger case series may be available in the near future to examine the prognostic significance.

As recommended for inclusion in pathological reports of pleural mesotheliomas, we also analyzed *p16/CDKN2A* deletion and PD-L1 status. The results of FISH analysis for *p16/CDKN2A* showed the worst prognosis when a homozygous deletion was detected. This finding confirms our previous work [6] and is consistent with the literature. The prognostic significance of *p16/CDKN2A* deletion in DMPM is well recognized [22], and it was suggested as a tool to help identify patients with a favorable outcome after multimodal treatments [23].

Positive PD-L1 immunostaining was detected in only one biphasic mesothelioma. PD-L1 tumor expression in DMPM was amply reported in the literature. One plausible explanation for this discrepancy is the small sample size of non-epithelioid cases, which are more commonly PD-L1 positive [24, 25]. This result is important and warrants in-depth investigation.

The contribution of Ki67 was introduced as a strong recommendation in the last PSOGI/EURACAN clinical practice guidelines for DMPM [7]. Ki67 was not strictly associated with survival of epithelioid mesotheliomas in our multivariate analysis. This topic remains controversial for several reasons. Although its prognostic value was suggested in the literature, there is no consensus on which scoring system should be used [12, 13, 26–28]. A two-tier system of categorization for Ki67 may be informative for prognosis as punctual values, but standardization of the methodology is mandatory. Digital imaging and virtual scoring may be helpful for this purpose. Its role deserves further study due to the uncertainty of the results.

Females showed lower overall survival than males in our study. This result contrasts with previous works [29] but it may be due to the limited number of female patients recruited. None of our cases were treated with combined-modality management, but complete cytoreduction, lack of lymph node metastasis, and low peritoneal cancer index (PCI) [13, 30–33] strictly correlated with significant improvement in survival in DMPM. An evaluation of histological parameters could further improve these results via better patient risk stratification.

The present study has some limitations. First, it was a retrospective study design. The relatively small sample size did not allow all morphological evaluations and statistical correlations. Second, PD-L1 was not available for all patients due to the lack of neoplastic cells in the residual tissue. However, the high number of epithelioid cases allowed a reliable evaluation of the feasibility of the grading categorization.

This study examined the prognostic significance of factors proposed for pleural mesothelioma in an extrapleural site. However, these results must be seen from a specific peritoneal perspective. The two entities are distinct but some histological features could provide a help in terms of morphological prognosis (in particular the nuclear grading system) and could provide a reliable standard for the diagnostic reporting. The collection of sufficient information for use in further multicentric studies would allow the prognostic value of each factor to be determined and validated, and answer unsolved questions to orient patient treatment in the right direction. A detailed report specifying additional morphological, immunohistochemical and molecular supportive data is desirable in this rare disease.

## Data Availability

The datasets used andanalyzed during the current study are available from the corresponding author on reasonable request.

## Abbreviations

DMPM: Diffuse malignant peritoneal mesothelioma
MM: Malignant mesothelioma
EURACAN: European Reference Network on Rare Adult Cancers
IASLC: International Association for the Study of Lung Cancer
WT1: Wilms’ Tumor 1
BAP1: BRCA1 associated protein 1
PD-L1: Programmed Death-Ligand 1
FISH: fluorescence in situ hybridization
